# Association of Iron Deficiency With the Severity of Heart Failure With Preserved Ejection Fraction: A Hospital-Based Comparative Observational Study From Bangladesh

**DOI:** 10.7759/cureus.113308

**Published:** 2026-07-24

**Authors:** Dewan Mushfiq Raihan, Mohammad Ullah, Hashan Mamunur Rahman, Md. Saifur Rahman

**Affiliations:** 1 Cardiology, Shaheed Ziaur Rahman Medical College Hospital, Bogura, BGD; 2 Cardiology, National Institute of Cardiovascular Diseases, Dhaka, BGD

**Keywords:** bangladesh, diastolic dysfunction, heart failure with preserved ejection fraction, iron deficiency, nt-probnp, nyha functional class

## Abstract

Background

Iron deficiency is a prevalent and clinically significant comorbidity in heart failure with preserved ejection fraction (HFpEF), yet local data from Bangladesh remain scarce. This study aimed to examine the association between iron deficiency and heart failure severity among patients with HFpEF attending a tertiary cardiac center in Dhaka.

Methods

This hospital-based comparative observational study enrolled 120 adult patients with HFpEF at Dhaka Medical College Hospital between July 2023 and June 2024. Patients were divided into two groups based on iron status: iron-deficient (Group I, n=60) and iron-replete (Group II, n=60). Iron deficiency was defined according to contemporary heart failure guidelines. Heart failure severity was assessed using the New York Heart Association (NYHA) functional classification, serum N-terminal pro-B-type natriuretic peptide (NT-proBNP) level, and echocardiographic diastolic dysfunction grading per the 2016 ASE/EACVI recommendations. Statistical analysis was performed using SPSS version 26.0 (IBM Corp., Armonk, USA), employing the independent-samples t-test for continuous variables and the chi-square or Fisher's exact test for categorical variables, as appropriate.

Results

Iron-deficient patients were significantly older (66.1±8.66 vs. 57.87±8.16 years; p < 0.001) and had substantially higher anaemia prevalence (63.3% vs. 18.3%; p<0.001). Iron deficiency was significantly associated with higher NYHA functional class (p=0.003), markedly elevated NT-proBNP levels (3256.75 ± 1124.30 vs. 2099.07 ± 987.50 pg/mL; p < 0.001), and more advanced diastolic dysfunction (p=0.028), with Grade 2 diastolic dysfunction predominating and Grade 3 diastolic dysfunction being more frequent in the iron-deficient group.

Conclusions

Iron deficiency was significantly associated with greater HFpEF severity across functional, biochemical, and echocardiographic domains, though baseline differences in age and anaemia may have contributed to this unadjusted association. These findings support further evaluation of routine iron status assessment in HFpEF management in Bangladesh, warranting confirmation through prospective, multicenter studies with multivariable adjustment.

## Introduction

Heart failure (HF) is a leading global cardiovascular public health crisis, affecting approximately 56.5 million people worldwide, with an age-standardized prevalence that rose from 647.9 to 682.7 per 100,000 between 1990 and 2021, and estimates suggest over 80 million individuals may be affected when broader diagnostic criteria are applied [1,2]. Heart failure with preserved ejection fraction (HFpEF) has emerged as the dominant HF phenotype, currently accounting for approximately 50% of all HF cases, with its prevalence expected to surpass that of heart failure with reduced ejection fraction (HFrEF) in the coming decades [3]. Despite a five-year survival below 50%, comparable to HFrEF and a rising hospitalization burden, HFpEF lacks disease-modifying therapies with proven mortality benefit, underscoring its formidable clinical challenge [3,4].

The pathophysiology of HFpEF reflects a systemic, multifactorial syndrome driven by common comorbidities such as obesity, hypertension, type 2 diabetes, and chronic kidney disease, which induce chronic low-grade inflammation, endothelial dysfunction, and oxidative stress [5]. These processes result in myocardial microvascular inflammation, reduced nitric oxide bioavailability, titin hypophosphorylation, and increased cardiomyocyte stiffness, producing the hallmark diastolic dysfunction, elevated filling pressures, and exercise intolerance characteristic of HFpEF [5]. Concurrent mitochondrial dysfunction further impairs cellular energy generation, explaining why HFpEF remains resistant to neurohormonal therapies that are effective in HFrEF [5].

Iron deficiency (ID) is an increasingly recognized and clinically important comorbidity in chronic HF. Iron is indispensable for oxygen transport, mitochondrial electron transport, and cellular energy metabolism; its deficiency directly impairs cardiac and skeletal muscle oxidative function, contributing to fatigue and exercise intolerance independent of anaemia [6]. ID affects 30-70% of HF patients, including approximately 50% of outpatients and 72-83% of those with acute decompensated HF, and is associated with higher NYHA functional class, elevated NT-proBNP, increased HF hospitalization, and greater mortality risk [7,8].

In HFpEF specifically, ID is highly prevalent - a systematic review estimated a pooled prevalence of 59% - and is associated with worsened diastolic dysfunction, impaired exercise capacity, poorer quality of life, and worse prognosis [9]. Intravenous ferric carboxymaltose (FCM) has demonstrated clinical benefits in HFrEF, supported by the AFFIRM-AHF trial and a meta-analysis of 14 RCTs [10,11]. However, evidence in HFpEF remains limited; the dedicated FAIR-HFpEF trial was stopped early after 39 patients, though it showed a meaningful improvement in six-minute walk test distance, leaving the therapeutic role of iron repletion in HFpEF inconclusive [12].

In low- and middle-income countries (LMICs) such as Bangladesh, cardiovascular disease (CVD) has recently surpassed infectious diseases as the leading cause of death, and Bangladesh experienced a 27.4% increase in overall CVD burden in recent decades [13]. Data from the GBD study (Global Burden of Diseases, Injuries, and Risk Factors Study) place Bangladesh's age-standardized HF prevalence at 275 per 100,000, with South Asia showing an increasing regional trend [14]. Despite this growing burden, local evidence characterizing ID in HFpEF patients remains virtually absent, and the distinct nutritional status and comorbidity profiles of Bangladeshi patients may differ substantially from Western cohorts [15]. Regional data are therefore essential for evidence-based screening and management. This comparative observational study aimed to examine the association between iron deficiency and heart failure severity among patients with HFpEF attending a tertiary cardiac center in Dhaka.

## Materials and methods

Study design and setting

This hospital-based comparative observational study was conducted in the Department of Cardiology, Dhaka Medical College Hospital (DMCH), Dhaka, Bangladesh, over a period of 12 months from July 2023 to June 2024.

Study population and sampling

Eligible patients with HFpEF were recruited using purposive fixed-quota sampling. The target sample of 120 participants was prespecified in the original study protocol based on the anticipated availability of eligible patients during the 12-month recruitment period and the feasibility of completing the required clinical, laboratory, and echocardiographic assessments. After eligibility assessment, iron status was determined, and recruitment continued within each iron-status stratum until the prespecified quota of 60 iron-deficient and 60 iron-replete participants was reached. The 1:1 allocation represented a deliberate quota for comparative analysis and did not constitute randomization or individual matching. Therefore, the equal group sizes did not represent the natural distribution of iron deficiency in the HFpEF population, and the study was not designed to estimate its prevalence. This sampling approach may also have introduced selection bias.

Inclusion and exclusion criteria

Patients aged >18 years were included if they fulfilled the predefined diagnostic criteria for HFpEF and provided written informed consent. Patients with significant valvular heart disease, infiltrative cardiomyopathy, hypertrophic cardiomyopathy, congenital heart disease, pericardial disease, atrial fibrillation, chronic obstructive pulmonary disease, renal failure with estimated glomerular filtration rate <60 mL/min/1.73 m², malignancy, chronic blood loss, recent iron therapy, or heart failure with improved ejection fraction were excluded.

Diagnostic criteria for HFpEF

Heart failure with preserved ejection fraction was diagnosed based on symptoms and/or signs of heart failure, left ventricular ejection fraction (LVEF) ≥50%, and objective evidence of elevated cardiac filling pressure, supported by elevated NT-proBNP and/or echocardiographic evidence of diastolic dysfunction or structural heart disease [16]. Echocardiographic evidence included abnormalities in mitral inflow pattern, average E/e′ ratio, left atrial volume index, septal and lateral e′ velocity, or tricuspid regurgitation velocity, assessed according to the 2016 American Society of Echocardiography/European Association of Cardiovascular Imaging (ASE/EACVI) recommendations [17].

Data collection procedure

After enrollment, detailed demographic and clinical information, including age, sex, cardiovascular risk factors, comorbidities, and medication history, were recorded using a semi-structured questionnaire. Symptoms and signs of heart failure were assessed clinically. Blood pressure and pulse were measured in all participants. Laboratory investigations included a complete blood count, a serum iron profile, and an NT-proBNP level. Iron profile assessment included serum iron, serum ferritin, and total iron-binding capacity (TIBC). Transferrin saturation (TSAT) was calculated by dividing serum iron by total iron-binding capacity and multiplying by 100. Hematological and biochemical analyses were performed in the Biochemistry Laboratory of Bangabandhu Sheikh Mujib Medical University using Atellica (Siemens Healthineers, Erlangen, Germany) and Alinity ci (Abbott Diagnostics, Lake County, USA) automated analyzers. Serum iron, ferritin, TIBC, and NT-proBNP were measured according to standard laboratory protocols. Details of analyzer manufacturer, assay platform, and reagent specifications were recorded from the laboratory reports. According to World Health Organization criteria, anaemia was defined as a hemoglobin concentration <12 g/dL in women and <13 g/dL in men. Iron deficiency was defined as serum ferritin <100 ng/mL or ferritin 100-299 ng/mL with transferrin saturation <20%, according to contemporary heart failure guidelines [18].

Echocardiographic assessment

All participants underwent transthoracic Doppler echocardiography using a commercially available ultrasound system with a 3.5 MHz transducer (Affinity 70C, Philips Healthcare, Amsterdam, Netherlands). Echocardiographic examinations were performed in the supine and left lateral decubitus positions using standard parasternal and apical views. Left ventricular ejection fraction was calculated using the modified Simpson’s method. Diastolic dysfunction was assessed according to the 2016 ASE/EACVI recommendations. Parameters evaluated included mitral inflow E/A ratio, average E/e′ ratio, left atrial volume index (LAVI), septal and lateral e′ velocity, and peak tricuspid regurgitation velocity. The key abnormal criteria included septal e′ velocity <7 cm/s, lateral e′ velocity <10 cm/s, average E/e′ ratio >14, LAVI >34 mL/m², and peak tricuspid regurgitation velocity >2.8 m/s. Diastolic dysfunction was graded as Grade I, Grade II, or Grade III according to mitral inflow pattern and supportive evidence of elevated left ventricular filling pressure, as recommended by the 2016 ASE/EACVI guideline [19].

Assessment of heart failure severity

Severity of HFpEF was assessed using New York Heart Association (NYHA) functional classification, NT-proBNP level, and echocardiographic grading of diastolic dysfunction. NYHA functional class was categorized from Class I to Class IV according to symptom severity and limitation of physical activity [20].

Statistical analysis

The Statistical Package for the Social Sciences (SPSS), version 26.0 (IBM Corp., Armonk, USA), was used for data analysis. Data were checked for completeness, consistency, and accuracy before analysis. Continuous variables were expressed as mean ± standard deviation, whereas categorical variables were presented as frequency and percentage. The independent-samples t-test was applied to compare continuous variables between the two groups. Categorical variables were compared using the chi-square test; however, Fisher's exact test was used when expected cell frequencies were less than 5. All between-group comparisons were unadjusted. A two-sided p-value of <0.05 was considered statistically significant.

Ethical assessment

Ethical approval for this study was obtained from the Ethical Committee of Dhaka Medical College, Dhaka, Bangladesh, before commencement of the study (Approval no: ERC-DMC/ECC/2023/203). The study was carried out in accordance with the ethical principles outlined in the Declaration of Helsinki. Written informed consent was obtained from all participants after explaining the objectives, procedures, potential benefits, and possible risks of the study. Privacy, confidentiality, and anonymity of all collected information were carefully preserved throughout the research process. Participants were informed that their participation was entirely voluntary and that they could withdraw from the study at any time without affecting their treatment or standard medical care.

## Results

Baseline demographic characteristics, cardiovascular risk factors, and anaemia prevalence of both groups are summarized in Table 1. The mean age of iron-deficient patients (Group I) was significantly higher than that of patients with normal iron status (Group II) (66.1 ± 8.66 years vs. 57.87 ± 8.16 years; p < 0.001). Female patients predominated in both groups, with 35 (58.3%) in Group I and 32 (53.3%) in Group II; however, this difference was not statistically significant (p=0.52). The prevalence of hypertension, diabetes mellitus, ischemic heart disease, and dyslipidaemia did not differ significantly between the two groups (all p>0.05). Anaemia was significantly more prevalent among iron-deficient patients, where 38 (63.3%) patients in Group I had anaemia compared with 11 (18.3%) patients in Group II (p<0.001).

**Table 1:** Comparison of baseline characteristics between Group I and Group II (n = 120). Group I = iron-deficient HFpEF patients; Group II = HFpEF patients with normal iron status; p values for categorical variables were calculated using the Chi-square test; for continuous variables, the independent samples t-test was applied. Significance threshold: p < 0.05; Values are presented as mean ± SD or number (%). df = degrees of freedom; φ = phi coefficient; HFpEF = heart failure with preserved ejection fraction

Characteristic	Group I frequency (%)	Group II frequency (%)	Test statistic	df	Effect size	p value
Age (years)						
Mean ± SD	66.1 ± 8.66	57.87 ± 8.16	t = 5.31	118	Cohen’s d = 0.97	< 0.001
Gender						
Male	25 (41.7)	28 (46.7)	χ² = 0.42	1	φ = 0.06	0.52
Female	35 (58.3)	32 (53.3)				
Cardiovascular risk factors						
Hypertension	47 (78.3)	49 (81.7)	χ² = 0.21	1	φ = 0.04	0.648
Diabetes mellitus	38 (63.3)	31 (51.7)	χ² = 1.67	1	φ = 0.12	0.196
Ischaemic heart disease	23 (38.3)	21 (35.0)	χ² = 0.14	1	φ = 0.03	0.704
Dyslipidaemia	19 (31.7)	17 (28.3)	χ² = 0.16	1	φ = 0.04	0.690
Anaemia status						
Present	38 (63.3)	11 (18.3)	χ² = 25.30	1	φ = 0.46	< 0.001
Absent	22 (36.7)	49 (81.7)				

Mean NT-proBNP concentration was significantly higher in iron-deficient patients (Group I: 3256.75 ± 1124.30 pg/mL) than in those with a normal iron status (Group II: 2099.07 ± 987.50 pg/mL; p < 0.001) (Table 2).

**Table 2:** Comparison of NT-proBNP between Group I and Group II (n = 120). Group I = iron-deficient HFpEF patients; Group II = HFpEF patients with normal iron status; p value calculated using the independent samples t-test; Values are presented as mean ± standard deviation. NT-proBNP, N-terminal pro-B-type natriuretic peptide; SD, standard deviation; df, degrees of freedom. NT-proBNP = N-terminal pro-B-type natriuretic peptide; HFpEF = heart failure with preserved ejection fraction

Variable	Group I Mean ± SD	Group II Mean ± SD	Test statistic	df	Effect size	p value
NT-proBNP (pg/mL)	3256.75 ± 1124.30	2099.07 ± 987.50	t = 5.99	118	Cohen’s d = 1.09	< 0.001

Iron deficiency was significantly associated with a higher NYHA functional class (p=0.003). Among Group I patients, 23 (38.3%) were classified as NYHA Class III and 4 (6.7%) as Class IV, indicating severe functional limitation. In contrast, 29 (48.3%) of Group II patients were in NYHA Class I, reflecting mild or absent symptomatic impairment. Diastolic dysfunction grading by transthoracic echocardiography, performed according to the 2016 ASE/EACVI recommendations, was significantly associated with iron deficiency (p=0.028). Grade 2 diastolic dysfunction was the most prevalent finding in Group I, with 29 (48.3%) patients, whereas Grade 1 was most common in Group II, with 38 (63.3%) patients. Grade 3 diastolic dysfunction was present in six (10.0%) of iron-deficient patients compared with only one (1.7%) of those with a normal iron status, indicating that more advanced diastolic dysfunction is significantly more frequent among iron-deficient HFpEF patients (Table 3).

**Table 3:** Association between iron deficiency and primary heart failure severity outcomes among HFpEF patients (n = 120) Group I = iron-deficient HFpEF patients; Group II = HFpEF patients with normal iron status. Diastolic dysfunction was graded per 2016 American Society of Echocardiography/European Association of Cardiovascular Imaging (ASE/EACVI) recommendations. p-values were calculated using Fisher's exact test. Significance threshold: p < 0.05. NYHA = New York Heart Association; HFpEF = heart failure with preserved ejection fraction

Outcome	Group I frequency (%)	Group II frequency (%)	p value
NYHA functional class			
Class I	14 (23.3)	29 (48.3)	0.003
Class II	19 (31.7)	21 (35.0)	
Class III	23 (38.3)	9 (15.0)	
Class IV	4 (6.7)	1 (1.7)	
Diastolic dysfunction grade			
Grade 1	25 (41.7)	38 (63.3)	0.028
Grade 2	29 (48.3)	21 (35.0)	
Grade 3	6 (10.0)	1 (1.7)	

## Discussion

This study examined the association between iron deficiency and heart failure severity in patients with HFpEF. Our findings revealed that iron deficiency was significantly linked to worse functional outcomes, including higher NYHA class, elevated NT-proBNP levels, and more advanced diastolic dysfunction. Iron-deficient patients were considerably more likely to present with Grade 2 or Grade 3 diastolic dysfunction, while iron deficiency was associated with echocardiographic and biomarker-defined disease severity in this HFpEF cohort.

One of the most notable observations in the present study was the significantly higher mean age among iron-deficient HFpEF patients compared to those with normal iron status. This finding aligns with evidence from previous investigations demonstrating that older patients with HF are disproportionately affected by iron deficiency, partly because ageing is accompanied by chronic low-grade systemic inflammation that upregulates hepcidin, the key iron-regulatory hormone, thereby impairing gastrointestinal iron absorption and promoting functional iron sequestration within the reticuloendothelial system [21]. A comprehensive review examining the interplay between inflammation, iron deficiency, and HF in older adults confirmed that hepcidin-mediated iron restriction is a prominent contributor to iron deficiency in elderly HF populations, independent of dietary insufficiency [22]. The substantially higher prevalence of anaemia among iron-deficient patients observed in the present study is consistent with the mechanistic understanding that prolonged or severe iron deficiency ultimately impairs erythropoiesis; indeed, Köseoğlu and Özlek reported comparable anaemia burden among iron-deficient HFpEF patients in a six-year follow-up cohort [9]. Crucially, however, a meaningful proportion of iron-deficient patients in the present study did not have anaemia, corroborating established evidence that iron deficiency exerts clinically significant effects on cardiac and skeletal muscle function through non-haematopoietic mechanisms, independent of haemoglobin concentration [6]. However, as age was not statistically adjusted for in this analysis, the extent to which the observed severity differences are attributable to iron deficiency independent of age cannot be determined from the present data.

Another important finding was the significant association between iron deficiency and both higher NYHA functional class and markedly elevated NT-proBNP levels. Iron-deficient patients were substantially more likely to exhibit severe functional limitation, whereas the majority of iron-replete patients demonstrated mild or absent symptoms. Similar findings have been reported in previous HF studies, demonstrating that the presence of iron deficiency, along with higher NYHA class and elevated NT-proBNP, predicted increased myocardial energy expenditure, underscoring the mechanistic link between iron status and functional cardiac severity [23]. In decompensated HF populations, iron-deficient patients consistently exhibited higher NT-proBNP levels and worse NYHA functional class compared to iron-replete counterparts, a pattern also observed in the present cohort [24]. Potential biological mechanisms that may contribute to this observed association include the essential role of iron in mitochondrial oxidative phosphorylation; iron deficiency may impair adenosine triphosphate (ATP) synthesis within cardiomyocytes and skeletal myocytes, potentially reducing contractile reserve and contributing to increased ventricular wall stress and natriuretic peptide release [25]. A systematic meta-analysis further confirmed that iron deficiency is associated with NYHA class severity and elevated NT-proBNP in HF patients, irrespective of ejection fraction, supporting the potential clinical relevance of the higher NT-proBNP levels observed among iron-deficient patients [8]. It should be noted that anaemia, which was significantly more prevalent among iron-deficient patients, may itself contribute to elevated NT-proBNP and worse NYHA class; the present analysis cannot fully disentangle the independent contribution of cellular iron deficiency from that of anaemia.

Findings from the present study further suggest a significant association between iron deficiency and the severity of diastolic dysfunction as assessed by the 2016 ASE/EACVI echocardiographic grading system. Iron-deficient patients were more likely to have Grade 2 or Grade 3 diastolic dysfunction, whereas Grade 1 predominated among those with normal iron status; thus, more advanced grades of diastolic dysfunction were observed more frequently in the iron-deficient group. The OptimEx-Clin subanalysis by Gevaert et al. was the first study to demonstrate a statistically significant association between iron parameters and echocardiographic indices of diastolic function in HFpEF patients, providing important precedent for the present findings [26]. Several potential biological mechanisms may help explain this observed relationship. Iron deficiency has been associated with mitochondrial dysfunction and oxidative stress within cardiomyocytes, which may impair energetic efficiency, reduce nitric oxide bioavailability, increase passive cardiomyocyte stiffness, and adversely affect diastolic relaxation [25,27]. A randomized controlled study by Mollace et al. demonstrated that intravenous ferric carboxymaltose significantly improved echocardiographic diastolic function parameters alongside reductions in oxidative stress biomarkers and improved endothelial reactivity; these findings provide experimental evidence that iron repletion may influence pathways related to diastolic function in HFpEF [28]. In contrast, the study by Barandiarán Aizpurua et al. involving 300 patients with HFpEF reported that iron deficiency was more strongly associated with prognosis than with exercise capacity itself, suggesting that its effects on diastolic function and haemodynamic burden may represent a mechanistic pathway distinct from its effects on skeletal myopathy [29].

This study is among the earliest from Bangladesh to examine the relationship between iron deficiency and HFpEF severity. Echocardiographic assessment followed the validated 2016 ASE/EACVI criteria, and standardized laboratory procedures were applied across both groups. However, the single-center setting and modest sample size may limit generalizability, while the comparative observational design precludes causal inference. Purposive fixed-quota sampling was used to enroll equal numbers of iron-deficient and iron-replete patients; participants were neither randomized nor matched, which may have introduced selection bias and prevents estimation of iron-deficiency prevalence. Significant differences in age and anaemia status were also present between groups. As multivariable regression and non-anaemic subgroup analyses were not performed, the independent contribution of iron deficiency could not be determined. Furthermore, the exclusion of patients with common HFpEF comorbidities, including atrial fibrillation, chronic obstructive pulmonary disease, and moderate-to-severe renal impairment, may limit the applicability of the findings to broader real-world HFpEF populations. Therefore, the findings should be interpreted as unadjusted, exploratory, and hypothesis-generating associations. Future multicenter studies with larger samples and appropriate adjusted analyses are warranted.

## Conclusions

The present study demonstrated a significant unadjusted association between iron deficiency and greater heart failure severity among HFpEF patients, reflected by higher NYHA functional class, markedly elevated NT-proBNP levels, and more advanced echocardiographic diastolic dysfunction. Anaemia was also substantially more frequent among iron-deficient patients. However, given the observed baseline imbalances in age and anaemia status between groups and the absence of multivariable adjustment, these findings should be considered hypothesis-generating rather than confirmatory of an independent effect of iron deficiency. Nonetheless, they underscore the potential clinical relevance of routine iron status assessment in HFpEF management, particularly in resource-limited settings such as Bangladesh. Further prospective, multicenter studies with larger sample sizes and multivariable adjustment are warranted to establish causal relationships, clarify the independent contribution of iron deficiency, and evaluate the therapeutic impact of iron repletion in this population.
